# Effects of benzalkonium chloride- or polyquad-preserved fixed combination glaucoma medications on human trabecular meshwork cells

**Published:** 2011-07-02

**Authors:** David A. Ammar, Malik Y. Kahook

**Affiliations:** University of Colorado Hospital Eye Center, University of Colorado Denver, Department of Ophthalmology, Aurora, CO

## Abstract

**Purpose:**

We investigated the potential short and long-term effects in cultured human trabecular meshwork (TM) cells of various topical glaucoma formulations containing different preservatives.

**Methods:**

We tested the fixed combination medications 0.004% travoprost plus 0.5% timolol preserved with either 0.015% benzalkonium chloride (BAK; DuoTrav®), or with 0.001% polyquad (PQ; DuoTrav^®^ BAK-free); and 0.005% latanoprost plus 0.5% timolol preserved with 0.020% BAK (Xalacom^®^). Also tested was a range of BAK concentrations (0.001%–0.020%) in balanced salt solution (BSS). Cells were treated for 25 min at 37 °C with solutions diluted 1:10 and 1:100 to mimic the reduced penetration of topical preparations to the anterior chamber. The percentage of live cells was determined immediately after treatment through the uptake of the fluorescent vital dye calcein-AM. To determine any long-term effects, we assayed release of matrix metalloproteinase 9 (MMP-9) and apoptosis 24 h after treatments.

**Results:**

BAK demonstrated a dose-dependent reduction in TM cell viability, ranging from 71±5% live cells at 0.001% BAK (diluted 1:10) to 33±3% live cells at 0.020% BAK (diluted 1:10). Travoprost (0.004%) plus 0.5% timolol preserved with 0.015% BAK had statistically fewer live TM cells (79±7%) than the same preparation preserved with 0.001% polyquad® (PQ; 93±1%; p<0.001). Latanoprost plus timolol preserved with 0.020% BAK (29±9% live cells) was similar to the 0.020% BAK (33±3%) treatment. However, travoprost plus timolol preserved in 0.015% BAK had significantly more live cells (83±12%) than the 1:10 dilution of 0.015% BAK (49±10%). We also found 0.020% BAK (diluted 1:100) resulted in elevated levels of extracellular MMP-9 at 24 h.

**Conclusions:**

These results demonstrate that the substitution of the preservative BAK from topical ophthalmic drugs results in greater in vitro viability of TM cells. Travoprost with timolol, but not latanoprost with timolol, countered some of the toxic BAK effects. BAK treatment appeared to cause elevated levels of MMP-9, a matrix metalloproteinase implicated in the pathogenesis of glaucoma.

## Introduction

One of the commonly prescribed classes of intraocular pressure (IOP) lowering agents are the prostaglandin analogs (PGAs). PGAs act primarily by enhancing uveoscleral outflow of aqueous humor, however PGAs also appear to act on the trabecular meshwork (TM) to facilitate aqueous humor (AH) outflow through the conventional outflow pathway [1-3]. The beta adrenergic receptor antagonist (β-blocker; BB) timolol, which reduces IOP by reducing AH production, is often combined with PGAs as a second-line treatment after initial PGA monotherapy has failed (PGA+BB). Use of topical ophthalmic formulations with two hypotensive agents in a single bottle (fixed combination drug therapy) is a cost effective way to treat glaucoma, simplifies the treatment regimen, and has the added benefit of decreasing the number of daily exposures to the medications and preservatives contained in most topical ophthalmic preparations.

Human and animal studies have shown that chronic topical glaucoma therapy associated with daily use can lead to alterations in tear film, damage and remodeling of the corneal surface, an increase in inflammatory cytokines, as well as other deleterious effects [4-9]. Acute exposure models using animals or cell culture systems demonstrate significant damage/death to cornea and conjunctival cells either immediately after exposure or within 24 h [10-14]. Some toxicity can be attributed to either of the active ingredients of the PGA+BB fixed combination therapy [14,15]. However, much of the ocular surface changes seen with chronic daily topical glaucoma therapy are associated with the commonly used preservative, benzalkonium chloride (BAK) [7,8,16].

It has long been known that BAK at antiseptic concentrations increases corneal permeability to hydrophilic agents [17]. While this can potentially increase delivery of topically applied drugs to the aqueous humor (AH) and ultimately the sites of AH production and AH outflow, it would also increase delivery of BAK itself. Little is known about the long-term effects of BAK on the TM endothelial cells that populate this conventional outflow pathway. The aim of this study was to compare the in vitro effects on cultured TM cells of three formulations of PGA+BB fixed combination therapies preserved with either BAK (varying concentrations) or the related cationic polymer polyquad (PQ: 0.001%). We assayed the effects of these agents diluted 1:10 and 1:100 to mimic the concentrations that reach the anterior chamber.

## Methods

### Test and control solutions for cell treatment

Topical ophthalmic preparations included 0.004% travoprost plus 0.5% timolol with 0.015% BAK (DuoTrav^®^; Alcon Laboratories, Inc., Fort Worth, TX), 0.004% travoprost plus 0.5% timolol with 0.001% PQ (DuoTrav^®^ BAK-free; Alcon), and 0.005% latanoprost plus 0.5% timolol with 0.020% BAK (Xalacom^®^; Pfizer Inc., New York, NY). Not all of these therapies are commercially available in the United States. Also tested were a range of BAK solutions (0.001%, 0.005%, 0.010%, 0.015%, and 0.020%) in balanced salt solution (BSS; Alcon). For viability assays, the live control solution was BSS and the dead control solution was a fixative solution containing 70% methanol and 0.2% saponin in BSS.

### Cell culture and treatment conditions

TM42, a primary human trabecular meshwork cell line, was grown in Dulbecco's Modified Eagle Medium (DMEM; Invitrogen, Carlsbad, CA) supplemented with 10% fetal bovine serum (FBS; Invitrogen) and 50 U/ml penicillin and 50/ml streptomycin (Invitrogen) on flasks and plates previously coated with 0.1% gelatin (Sigma-Aldrich, St. Louis, MO) for 2 h at 37 °C. For 96-well assays, approximately 5×10^4^ TM cells were plated into each well of a coated 96-well tray. Fluorescent assays were plated in black opaque plates (Cellstar; Greiner Bio-One North America Inc., Monroe, NC), while absorbance-based assays were plated in clear plates (Cellstar). Cells were assayed upon reaching confluence, usually between 2 and 3 days post-plating. For assays, culture media from TM cells was removed by aspiration and exposed to 100 μl of test or control solutions (diluted 1:10 or 1:100 in serum-free DMEM) for 25 min at 37 °C and 5% CO_2_.

### Cell viability assays

The LIVE/DEAD^®^ Viability/Cytotoxicity Kit for mammalian cells (Invitrogen) was used to assay cell death immediately after treatment with test solutions. In this vital stain system, the Calcein-AM ‘LIVE’ stain accumulates in healthy cells due to non-specific cellular esterase activity that cleaves the cell-permeable ‘AM’ moiety from the dye. After 25 min of exposure, test and control dilutions were aspirated and replaced with a 100 μl solution of 2 μM Calcein-AM in Dulbecco's Phosphate-Buffered Saline without Calcium or Magnesium (D-PBS; Invitrogen). Uptake of fluorescent dye was quantified after 20 min in a Synergy™ 4 Multi-Mode Microplate Reader using the Gen5™ Reader Control and Data Analysis Software using the band-pass filter setting (BioTek, Winooski, VT). Live cells were quantified by determining the Calcein fluorescence (F_528_) collected at 528±20 nm from an excitation of 485±20 nm. Fluorescent data were analyzed as outlined in the manufacturer’s instructions. F_528_ fluorescence was normalized to the F_528_ fluorescence of the BSS-treated live controls (100% live).

The Vybrant Cytotoxicity Assay Kit (Invitrogen) was used to determine the death of TM cells cultured for 24 h after exposure to test and control solutions (diluted 1:100). This kit assays the release into the cell culture media of the exclusively cytosolic enzyme glucose 6-phosphate dehydrogenase (G6PD) using a fluorescent substrate. G6PD is an essential enzyme present in all cells and since it remains active for at least 24 h after cell death, extracellular G6PD activity correlates to the number of cell that have died. After 25 min of exposure, test and control solutions were aspirated and replaced with 100 µl of culture media. At 24 h, media was removed from the TM cells and saved for G6PD and matrix metalloproteinase assays (below). G6PD levels were quantified as outlined in the manufacturer’s instructions using 50 µl of TM cell culture supernatant. Briefly, G6PD-dependent production of resorufin was determined fluorescently in a Synergy™ 4 Multi-Mode Microplate Reader with excitation at 563 nm and emission collected at 587 nm. Fluorescent data were corrected by subtraction of fluorescence from no protein blanks, and normalized to fluorescence from lysed TM cells (% total G6DP).

### Matrix metalloproteinase assays

The colorimetric MMP-9 Human ELISA Kit (Invitrogen) was used to determine amount of matrix metalloproteinase 9 (MMP9) released into the extracellular media in TM cells cultured for 24 h after a 25 min exposure to test and control solutions (diluted 1:100). MMP-9 levels were quantified as outlined in the manufacturer’s instructions using 40 µl of TM cell culture supernatant (isolated as described above), with ELISA absorbance measurements performed at 450 nm using a Synergy™ 4 Multi-Mode Microplate Reader and compared to a MMP-9 standard curve.

### Apoptosis measurements

The appearance of the apoptosis marker (activated caspase-3) was determined 24 h after exposure to test solutions, cells undergoing apoptosis were detected by immunofluorescence using an antibody for cleaved caspase-3. Activation of caspase-3 requires a proteolytic processing of the inactive pro-enzyme; the antibody used only recognizes this cleaved (active) form and not the full-length form. TM cells were plated onto gelatin coated glass coverslips and treated as described above. After 25 min exposure, test and control solutions (diluted 1:100) were aspirated and replaced with culture media. At 24 h, cells were fixed, permeabilized and stained using an Alexa Fluor 488-conjugated antibody specific for cleaved Caspase-3 (Asp175; Cell Signaling, Beverly MA). Fluorescence imaging was performed using a Nikon Eclipse 80i microscope (Nikon, Melville, NY) equipped with a Nikon DS-Qi1 monochrome 1.5 megapixel CCD camera and a Nikon 10×/0.30 Plan Fluor objective. For each exposure, five fields of cells were photographed for counting of caspase-3 staining (ranging from 20 to 50 cells/field).

### Data and statistical analysis

The two cell viability assays were performed in two independent experiments in triplicate (n=6). MMP-9 assays were performed in duplicate. Five different field of views were counted for each exposure in the apoptosis assays (n=5). Data were reported as the mean±standard deviation. Statistical analysis was performed using the *t*-test (Excel, Microsoft; Redmond WA); the level of significance was set at p<0.05.

## Results

### Toxicity of BAK alone

**Figure 1:**
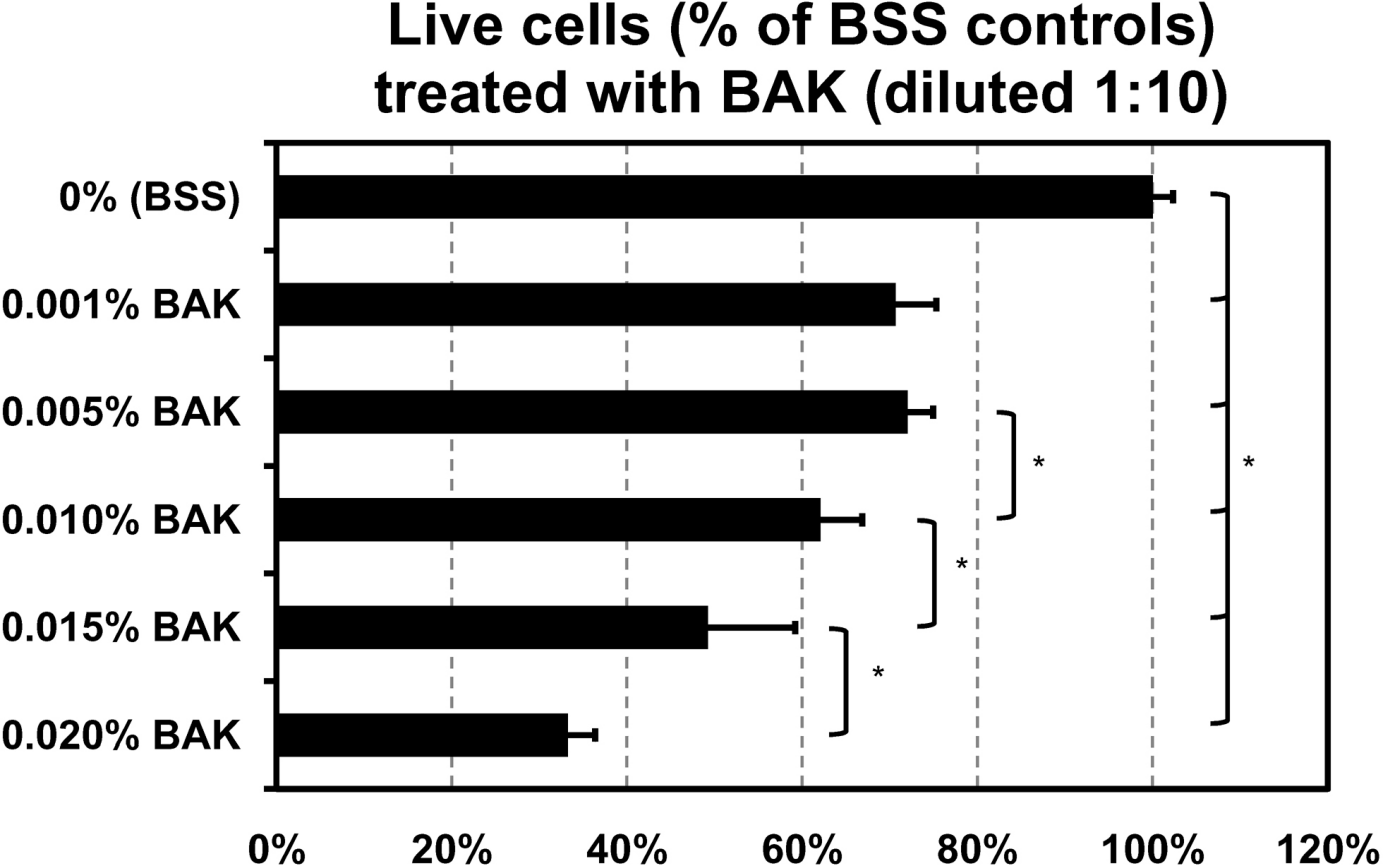
Percent of live trabecular meshwork (TM) cells after a 25 min exposure to various concentrations of benzalkonium chloride (BAK diluted 1:10 in serum-free media). The number of live cells was normalized to the number of live cells in BSS-treated controls. Data are reported as the mean±standard deviation of n=6 replicates. All concentrations of BAK were significantly different (p<0.05) from BSS-treated controls. There is a dose-dependent decrease in cell viability above 0.005% BAK (diluted 1:10). *=p<0.05.

TM cells showed a dose-dependent decrease in cell viability after exposure to BAK for 25 min when assayed immediately after exposure (Figure 1). All concentrations of diluted BAK were significantly different (p<0.05) from BSS-treated controls. There was no significant difference in cell viability between 1:10 dilutions of 0.001% and 0.005% BAK. We observed statistically significant differences in TM cell viability (*, p<0.05) among 1:10 dilutions of 0.005% BAK (72±3% live), 0.010% BAK (62±5% live), 0.015% BAK (49±10% live), and 0.020% BAK (33±3% live).

### TM cell survival with PGAs+BB preserved with BAK

**Figure 2:**
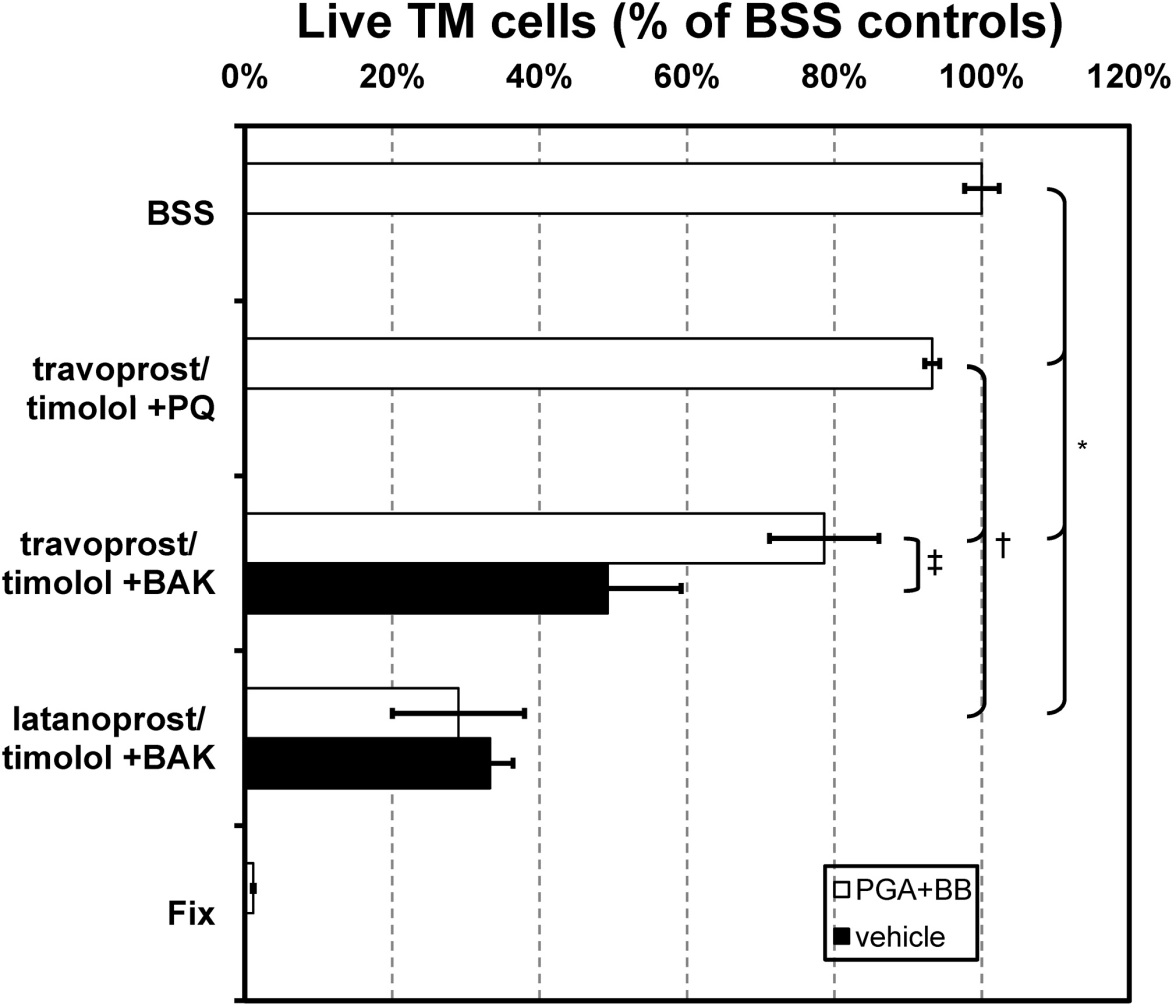
Percent of live trabecular meshwork (TM) cells after a 25 min exposure to various commercially available prostaglandin analogs (diluted 1:10 in serum-free media). The number of live cells was normalized to the number of live cells in BSS-treated controls. Data are reported as the mean±standard deviation of n=6 replicates. *=p<0.05 versus BSS control; †=p<0.05 versus travoprost/timolol + PQ; ‡=p<0.05 versus BAK alone.

TM cells exposed to 1:10 dilutions of both PGAs+BB formulations containing BAK had statistically significant fewer live cells compared to BSS controls (Figure 2, *). Comparing the two BAK-preserved formulations of PGA+BB, exposure to diluted travoprost+timolol with 0.015% BAK resulted in significantly more live TM cells (79±7%) than diluted latanoprost+timolol with 0.020% BAK (29%±9%, p<0.05). As shown in Figure 2 (‡), TM cells exposed to diluted travoprost+timolol with 0.015% BAK (79±7%) also had significantly more live cells compared to diluted 0.015% BAK (49±10% live cells). Cultures exposed to either diluted latanoprost+timolol with 0.020% BAK or diluted 0.020% BAK had similar survival rates (29±9% live cells versus 33±3%, p=0.29).

### TM cell survival with PGA+BB preserved with PQ

Travoprost+timolol with PQ diluted 1:10 caused a slight but significant reduction in the number of live TM cells (93%±1%) compared to BSS exposure (Figure 2, *), but had significantly more live cells compared to the same PGA+BB formulation containing 0.015% BAK (79±7%, Figure 2, †).

### TM cell survival with PGA+BB at 24 h

To determine the long-term cytotoxic effects in TM cells, cultures were exposed to a lower amount of test and control solutions (diluted 1:100) in serum-free media. TM cells were then returned to culture media and assayed after 24 h. Any TM cells which die over this period would release into the extracellular media the exclusively cytosolic enzyme glucose 6-phosphate dehydrogenase (G6PD). We found no significant differences between cultures exposed of test and control solutions when compared to BSS controls (Table 1).

**Table 1:** Release of Glucose 6-phosphate Dehydrogenase (G6PD) by trabecular meshwork at 24 h.

**PGA+BB formulations (dil. 1:100)**	**% Total G6PD**
Travoprost+timolol with PQ	3.0%±1.5%
Travoprost+timolol with BAK	2.1%±0.1%
**Original % BAK (dil. 1:100)**	**% Total G6PD**
0% (BSS)	1.6%±0.6%
0.001% BAK	2.1%±0.8%
0.005% BAK	1.8%±0.3%
0.010% BAK	1.8%±0.3%
0.015% BAK	2.2%±0.3%
0.020% BAK	2.5%±0.2%

TM cells were treated with test and control solutions (diluted 1:100) for 25 min, and then returned to fresh culture media. Media was assayed at 24 h (n=6), data were normalized to the amount of G6PD in lysed trabecular meshwork cells (100% G6PD, and expressed as mean±SD. No significant changes were noted from BSS control.

### TM cell survival with PGA+BB at 24 h

The release of matrix-metalloproteinase 9 (MMP-9) into the extracellular media was assayed alongside G6PD release. Using an assay with a minimum sensitivity of 10 pg/mL, we could not reproducibly detect MMP-9 in TM cells exposed to any of the PGAs+BB formulations (diluted 1:100). Among the BAK solutions, we could only reproducibly detect MMP-9 in cells treated with 1:100 dilution of 0.020% (33.4±8.8 pg/ml, n=2).

### TM cell apoptosis with PGA+BB at 24 h

Apoptosis was determined in TM cells 24 h after exposure to test and control solutions (diluted 1:100). We found an inverse relationship between BAK concentrations and the number of apoptotic TM cells (Table 2). When exposed to 0.001% BAK (diluted 1:100), we counted 44.5%±8.1% apoptotic TM cells compared to 15.9%±6.5% after exposure to 0.005% BAK (diluted 1:100), 12.8%±6.8% after exposure to 0.010% BAK (diluted 1:100), and 13.2%±6.0% after exposure to 0.015% BAK (diluted 1:100). TM cells exposed to 0.020% BAK (diluted 1:100) showed no statistical increase in apoptosis compared to BSS controls (5.8%±3.5% versus 4.4%±2.5%, respectively). This effect of increasing apoptosis with exposure to reducing concentrations of BAK is similar to that reported in conjunctival cells [18].

**Table 2:** Cleaved caspase-3 positive trabecular meshwork cells at 24 h.

**PGA+BB formulations (dil. 1:100)**	**% Apoptotic cells**
Travoprost+timolol with PQ	15.3%±6.3%*
Travoprost+timolol with BAK	18.7%±4.5%*
Latanoprost+timolol with BAK	11.9%±4.6%*†‡
**Original % BAK (dil. 1:100)**	**% Apoptotic cells**
0% (BSS)	4.4%±2.5%
0.001% BAK	44.5%±8.1%*
0.005% BAK	15.9%±6.5%*
0.010% BAK	12.8%±6.8%*
0.015% BAK	13.2%±6.0%*
0.020% BAK	5.8%±3.5%

TM cells were treated with test and control solutions (diluted 1:100) for 25 min, and then returned to fresh culture media. Cells were fixed and immunostained for cleaved caspase-3 at 24 h. Data are reported as the percent of cleaved caspase-3 positive cells ±SD (n=5). *p<0.05 versus BSS; †=p<0.05 versus travoprost+timolol with BAK; ‡=p<0.05 versus BAK alone.

TM cells exposed to any of the PGAs+BB formulations (diluted 1:100) had statistically more apoptotic TM cells compared to BSS controls (Table 2, *). Replacement of PQ with BAK in the travoprost+timolol fixed combination therapy has a small but not significant effect on apoptosis. Exposure of TM cells to diluted latanoprost+timolol with BAK resulted in significantly fewer apoptotic cells (11.9%±4.6%) than exposure to either diluted travoprost+timolol with BAK (18.7%±4.5%, Table 2, †) or diluted travoprost+timolol with PQ (15.3%±6.3%, Table 2). Since lower concentrations of BAK result in greater apoptosis of TM cells by some unknown mechanism, the difference seen here may be due solely to the lower BAK concentration in the latanoprost+timolol formulation. However, since diluted latanoprost+timolol with BAK caused significantly more apoptosis than the corresponding amount of BAK (diluted 0.020%, Table 2, ‡), this suggests that the increase in apoptosis is due to one of the active ingredients and not the preservative.

## Discussion

The current study examined the effects of different dilutions of commonly used topical ophthalmic medications either immediately after exposure or after 24 h. For short-term viability, we used vital dyes to distinguish live cells from dead which has proven to be a useful assay to determine the cytotoxicity of topical ophthalmic medications in cell culture systems that model acute exposure [10,13,14,19]. At a dilution of 1:10, we determined that replacement of preservative BAK with PQ in topical fixed combination therapies (PGA+BB) significantly increased the number of live TM cells when assayed immediately after exposure. When diluted to 1:100, we found no difference in the number of dead cells among any of the PGA+BB and BAK solutions. Exposure to concentrations of BAK commonly used as preservatives in topical ophthalmic preparations (diluted 1:10) are toxic to TM cells in a direct dose–response relationship when assayed immediately after exposure. In fixed combination therapies preserved with BAK, we found that exposure to travoprost+timolol resulted in greater percentage of live cells than latanoprost+timolol. We believe that some of this greater survival can be attributed to the lower concentration of BAK. However, we found that an inverse relationship between exposure to BAK diluted 1:100 and TM cell apoptosis at 24 h. The latanoprost+timolol exposure had fewer apoptotic cells than the travoprost+timolol exposure. This is likely due to the higher concentration of BAK in the latanoprost+timolol formulation. Our results mirror the apoptotic effect of BAK seen by others in conjunctival cells, in which BAK caused immediate necrosis at high concentrations, but apoptosis after treatment with low concentrations [18]. These researchers concluded that BAK caused immediate necrosis at high concentrations, but apoptosis after treatment with low concentrations. Currently, we have no explanation for why apoptosis is seen only at the lowest concentrations of BAK.

We also determined that exposure to 0.020% BAK (diluted 1:100) increased MMP-9 release by TM cells over 24 h, but not with exposure to diluted PGA+BB formulations preserved with BAK. The detection of MMP-9 in the TM can be problematic, and may depend on the method used for detection. One group reported near zero levels of *MMP-9* mRNA in cultured TM cells [20] while other groups have detected MMP-9 enzymatic activity in cultured TM cells cultures [21]. Increased MMP-9 activity was reported in TM organ cultures exposed to latanoprost at concentrations 20-fold higher than that used here [22]. In contrast, Oh et al. [20] found no increase in expression MMP-9 in TM cell cultures exposed to concentrations latanoprost similar to that used here. However, to our knowledge, no group has examined MMP-9 in TM cells exposure to diluted BAK alone. In this light, the effect of MMP-9 on the collagen matrix of the TM region is a question for future studies.

In our short-term viability assays, it is notable that diluted travoprost+timolol with 0.015% BAK performed better than 0.015% BAK alone, reflecting the protective effect that some prostaglandins demonstrate against the toxic effect of BAK in ocular surface epithelia [23]. A protective effect was not seen with latanoprost+timolol with 0.020% BAK in short-term viability assays; this result is in agreement with our previous cytotoxic assays in corneal and conjunctival using this vital dye system [24], but these results are in contrast to Guenoun et al. [23] who found a protective effect with both prostaglandins against BAK-induced toxicity in corneal and conjunctival cells.

Benzalkonium chloride (BAK) is the most commonly used preservative in topical glaucoma medications [25]. BAK is a quaternary ammonium compound that acts as a detergent, disrupting bacterial cell membranes and ultimately killing them. There is a broad consensus that chronic use of topical glaucoma medications is associated with ocular surface changes in both the cornea and conjunctiva, and several studies have linked these effects to the BAK content, both in vitro and in vivo, on the corneal and conjunctival epithelium and stroma [6,10,15,23,26-28]. While the antiseptic concentrations of BAK are cytotoxic, it is important to also consider the toxicity of the active ingredients. A100-fold dilution of a therapeutic dose of PGAs can increase the release of cytosolic proteins from corneal endothelial cells, while a 1:100 dilution of 0.01% BAK does not [14]. Similarly, undiluted BB in a preservative-free formulation causes a 40%–60% reduction in viability of human conjunctival cells [15].

The choice of BAK concentration and dosing regimen for an experimental animal model can greatly affect the outcome of BAK. For example, single daily dosing in rabbits for 30 days with 0.010% BAK showed minimal damage to corneal endothelial cells [29], but single daily dosing of PGA plus 0.020% BAK did show a reduction in goblet cell density and increase in inflammatory cells [7,30]. In contrast, instilling multiple doses over a short period of time (the Draize model), has shown changes to the ocular surface within one day, causing corneal/conjunctival cellular damage and an increase in inflammatory markers [11]. Therefore it is important to balance exposure time and concentration when evaluating the biologic effect of topical ophthalmic medications. While our exposure model does not model the repetitive exposure to topical ocular drugs experienced in vivo, the lower concentration of exposure is a good model for the accumulation of drug and preservative in the AH, and is a first a step in understanding the role of glaucoma therapy in ocular surface disease.

In conclusion, our findings argue that replacement of BAK with an alternate preservative (PQ) is potentially beneficial for TM cell viability. Since glaucoma is a chronic disease, it is important to consider the consequences of long-term exposure to both the anti-hypotensive agent as well as the included preservative. Studies in rabbits and humans demonstrate penetration of PGAs to the AH to be less than 1/1000th of the concentration applied [31,32]. Topical BAK has been shown to increased fluorescein permeability in rabbit cornea [17], and reduces the expression of the tight-junction protein occludin in a corneal cell culture system [33]. These findings, combined with the fact that BAK is itself absorbed and accumulates into ocular tissues [34], imply that the concentration of BAK in the AH may be much higher than the PGA levels. Since prior studies have shown that brief treatment of cultured TM cells with BAK can increase apoptotic cell markers and significantly decrease cell growth at levels 1/100th of that used as a preservative [35,36], the deleterious effect of BAK on TM cells may be an underappreciated concern. Furthermore, since the number of live TM cells within the meshwork was found to be statistically lower in patients with primary open-angle glaucoma [37], maintaining TM cell number should be as important a metric of disease treatment as intraocular pressure. The clinical implications of our findings suggest that long-term use of fixed combination therapies containing BAK could damage the structures within the TM by reducing the number of healthy TM cells. Therefore, replacement of BAK with PQ within topical glaucoma therapies may slow or prevent further damage to the TM region of the eye. This study also raises the question of whether activation of MMP-9 by BAK plays a positive or negative role in fluid flow through the TM. Additional in vitro studies are needed, and in vivo animal assays will need to be performed to ascertain the true clinical implications of our findings for patients treated chronically with fixed combination topical ophthalmic preparations.
